# Effects of Therapeutic Exercise Intensity on Cerebral Palsy Outcomes: A Systematic Review With Meta-Regression of Randomized Clinical Trials

**DOI:** 10.3389/fneur.2019.00657

**Published:** 2019-06-21

**Authors:** Che-Wei Hsu, Yi-No Kang, Sung-Hui Tseng

**Affiliations:** ^1^Department of Physical Medicine and Rehabilitation, Taipei Medical University Hospital, Taipei, Taiwan; ^2^Evidence-Based Medicine Center, Wan Fang Hospital, Taipei Medical University, Taipei, Taiwan; ^3^Department of Physical Medicine and Rehabilitation, School of Medicine, College of Medicine, Taipei Medical University, Taipei, Taiwan

**Keywords:** cerebral palsy, physical therapy, exercise intensity, gross motor function, metaregression

## Abstract

**Background and Objective:** Intensive physical therapy or exercise has been associated with favorable cerebral palsy (CP) outcomes, but few studies have investigated the effects of exercise intensity on the improvement in CP outcomes. In this study, we assessed the effects of intensive exercise-based therapy on improvement in gross motor function in children with CP.

**Methods:** We searched three databases for randomized clinical trials evaluating the effects of therapeutic exercise training by using Gross Motor Function Measurement (GMFM) 66 and 88 among children with CP. Studies that used interventions in addition to therapeutic exercise were excluded from the present meta-analysis. Exercise intensity was defined using the number of training hours per day and duration of intervention (in weeks). The effects of the number of daily training hours and program duration on GMFM improvement were evaluated using meta-regression.

**Results:** The comprehensive search returned 270 references, and 13 of 270 references met our eligibility criteria. The 13 trials recruited 412 children with CP. These trials measured motor improvements by using GMFM-66 (*n* = 8) and GMFM-88 (*n* = 5). The GMFM scores in the children who received the therapeutic intervention did not show significantly greater improvement than those of the children who received standard care. Meta-regression analysis revealed that the improvement in GMFM scores was positively associated with the number of daily training hours (point estimate = 0.549; *p* = 0.031) and program duration (point estimate = 0.067; *p* = 0.075).

**Discussion and Conclusions:** Intensive physical exercise improved CP outcomes in the intervention and standard therapy groups. The duration of therapeutic intervention improved CP outcomes among the children who received the therapeutic intervention, while an increase in the number of daily training hours improved in CP outcomes in the children who received standard therapy.

## Introduction

Cerebral palsy (CP), resulting from a non-progressive lesion in the immature brain, is among the most burdensome childhood disorders of movement control and posture (1). Walking is extremely crucial for independent mobility, performance of daily activities, and social participation as well as for maintaining quality of life. The objective of rehabilitation and therapeutic interventions for children with CP is to develop their ability to walk (2). Many therapeutic exercises have been designed to improve activity levels in children with CP for enhancing their mobility-related participation (3, 4).

Therapeutic exercise programs for children with CP are complex in terms of type, frequency, intensity, duration, and mode of delivery. Intensive physical therapy has been reported to improve functional outcomes. Although constraint-induced movement therapy (CIMT) trials and reviews have provided evidence suggesting that the effectiveness of CIMT for arm function in children with CP in intensive exercise programs is higher than the effectiveness in conventional therapy (5–7); however, whether intensive therapy is associated with an increase in gross motor function remains inconclusive. A systematic review that defined intensive intervention as “training more than two times per week” did not provide robust evidence of the effects of intensive training on gross motor function through meta-analysis because of heterogeneity in the interventions and outcomes in the included studies (8). However, in another meta-analysis, which included four randomized clinical trials (RCT), intensive intervention was defined as “training more than three times per week,” and the duration of the training programs ranged from 2 weeks to 6 months. In the aforementioned meta-analysis, the Gross Motor Function Measurement (GMFM) score was higher by 1.32 (95% confidence interval [CI] 0.55–2.10) in the intensive-training group than in the non-intensive-training group (9). The optimal intensity physical therapy for effective improvement in gross motor function remains undefined.

The definition of intensive physical training for children differs considerably from that for adults. For instance, 11 sessions per week over 4 weeks to 6.5 h per day of intervention over 13 days are considered intensive physical training programs for children (10). The intensity of physical therapy should receive sufficient attention in children with CP in value-based care because high-intensity physical therapy usually requires higher levels of determination and compliance from patients than standard physical therapy does. To achieve the goals of various types of exercise programs designed to improve the physical function in children with CP who are motivated to improve their gross motor function, evidence-based assessment of the effectiveness of exercise programs and clear training schedules of available treatment options are essential. We hypothesized that defined daily dose (number of daily training hours) and total duration of the exercise program are positively correlated with improvement in gross motor function in different types of interventions. Because no consensus has been reached currently regarding the optimal number of daily training hours and optimal duration of intervention, we investigated the relationship between the intensity of exercise-based therapy and improvement in gross motor function outcomes in children with CP through systematic review and meta-analysis.

## Methods

According to the Preferred Reporting Items for Systematic Reviews and Meta-Analyses (PRISMA) guidelines, we presented our evidence selection, risk of bias assessment, and meta-analysis.

### Search Strategy

All available years of data in MEDLINE, Embase, and Cochrane Library (including Cochrane Central Register of Controlled Trials, CENTRAL) were searched for potential references. The search strategy used free text word retrieval and subject headings adapted for each database as well as relevant key words such as “cerebral palsy” and “gross motor function measurement” with the filter for article type set on “randomized clinical trial (RCT).” An updated search was conducted in December 2018.

### Selection Criteria

Studies were included in the meta-analysis if the mean age of the population of patients with CP was <18 years, the effects of therapeutic interventions on gross motor function were evaluated using GMFM, the studies were RCTs, and they were published in peer-reviewed journals before December 2018. Studies were excluded if patient allocation was non-randomized; the intervention involved unique equipment or technology, pharmacological therapy, surgery, injections of botulinum toxin-A, and hippotherapy or passive interventions, such as hydrotherapy, laser, reflexology, and orthoses; or did not clearly mention the duration and frequency of intervention in both arms. Both GMFM-88 and GMFM-66 are validated tools and represent two of the most frequently used tools for assessment of functional motor ability in children with CP (11).

### Selection of Studies and Data Extraction

Two reviewers independently assessed all the studies at different steps in study selection and data extraction (i.e., study selection, data extraction, and risk of bias evaluation). Any disagreements between the reviewers in these processes were resolved through discussions with the authors. The titles and abstracts of all retrieved references were screened. The full texts of relevant publications were reviewed, and the studies were included if they met the inclusion criteria. The data from the included studies were extracted using a piloted data extraction form, which included information on the study population, design, interventions, comparison, outcome measures, and results.

### Risk of Bias

The risk of bias tool includes the following items: sequence generation, allocation concealment, integrity of blinding, completeness of outcome data, selective reporting, and other potential sources of bias. The items in the risk of bias assessment were classified according to the extent to which bias was prevented and were rated as having a low, high, or unclear risk of bias. An overall assessment of the risk of bias was assigned to each included study as suggested in the Cochrane Handbook. If five items received a low-risk rating within a study, the study was assigned an overall low risk of bias. The results of a study with a low risk of bias are unlikely to be affected by bias.

### Data Synthesis and Analysis

The comparisons of interest were GMFM outcomes and intervention intensity. We extracted the available total or combined scores of GMFM-88 and GMFM-66 or the individual scores (%) obtained using GMFM-88 and GMFM-66 from the trials. We defined intensity of intervention in terms of the duration (in weeks) of therapy and number of daily training hours. Specifically, we converted the duration and frequency of intervention described in the trials into daily training hours (h/d) by dividing the total number of training hours by the duration of the entire training period. Because people typically work 5 days per week and 4 weeks per month, we used 5 days to define 1 week and 4 weeks to define 1 month while converting data from the included studies. For example, when an intervention involved 1 h/d three times per week for 12 weeks, the number of daily training hours would be calculated as 1 (h/d) × 3 (times per week)/5 d = 1.1 h/d. Standardized mean differences (SMDs) were computed for pooling the outcomes from the GMFM-66 and the GMFM-88. Mean differences were used for outcomes obtained with the same measuring tool. Metaregression was applied to analyze the relationship between training intensity and improvement in gross motor outcomes. Comprehensive Meta-Analysis Software was used to compute SMDs, perform metaregression analysis, and summarize statistically randomized controlled data if the included studies were comparable in types of training, intensity of training, and clearly defined outcomes. A random effects model was used to account for pooling effects caused by clinical heterogeneity of the included studies. Double-data entries were performed. We aimed to examine characteristics that may have contributed to explain the variations in effects.

## Results

**Figure 1:**
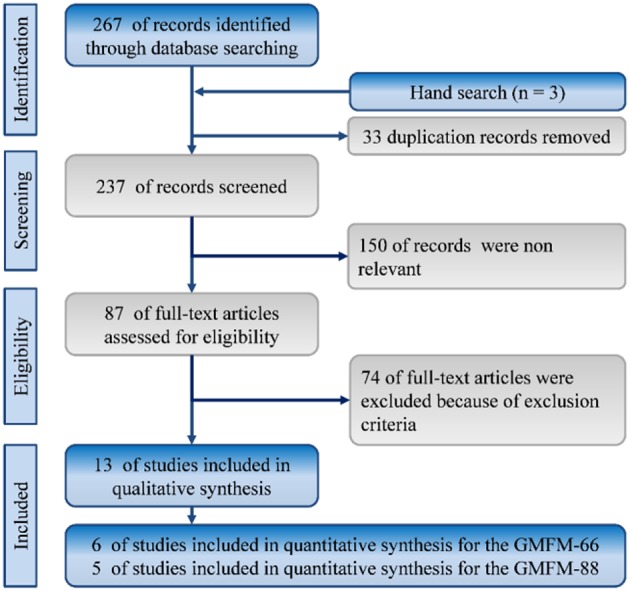
Flowchart of the systematic review and meta-analysis according to PRISMA guidelines. RCT: randomized controlled trials.

In total, 270 references were identified from the databases of MEDLINE and Embase. The titles and abstracts of these articles were screened by CWH and SHT. After duplicate studies were removed, we screened 237 records for titles and abstracts, and 87 articles were selected for full-text assessment. Finally, 13 studies met our inclusion criteria. Figure 1 depicts the selection process with reasons for exclusion.

### Characteristics and Quality of Included Studies

The 13 trials that met the eligibility criteria for analysis are listed in Table 1. All the included studies were RCTs. Overall, the 13 RCTs recruited 412 children with CP. The age range of the children was 1–17 years. The context of interventions involved between different techniques, such as motor learning coaching or task-oriented treatment vs. neurodevelopmental treatment (NDT) (12–14), specific techniques vs. standard therapy (14–18), static exercise bike vs. standard care, treadmill vs. standard care (19, 20), intermittent vs. continuous schedules (21), and intensive therapy vs. non-intensive therapy (22, 23). Seven of these studies had specified the participants' Gross Motor Function Classification System (GMFCS) level, but four of these studies included only children with walking ability (level I–III). Most trials showed improvements in GMFM scores during the treatment period in both the groups (12, 15, 16, 19, 21, 24). Interventions that were administered less than three times per week are usually considered non-intensive (8). Only six trials met the criterion of duration of treatment (12, 15, 16, 21, 24). Only In one study that assessed the effect of intervention intensity, the control group had a shorter program duration than the intervention group (23). Experimental duration ranged from 2 (22) to 30 (21) weeks. The weekly frequency ranged from three times per week (12) to everyday (15). The duration of a single session ranged from 0.5 (13, 19) to 2 (15) h. Daily training hours (daily dose) in both arms in these two studies were 0.24 and 6.5 h/d. The quality of the RCTs is shown in Supplementary Figure 1.

Table 1Characteristics of patients in the included studies.

**Table:** 

**Study**	**Country**	**Year**	**Sample**	**Type**	**Severity**	**Age (y)**
Arai	Japan	2014	16	Bil spastic	III–IV^+^	4–9
Bar-haim	Israel	2010	72	Spastic/Diplegia, Quadriplegia	II–III	8.8
Bower	UK	1996	44	Quadriplegic	Non-specific	3–11
Bryant	UK	2013	35	Dyskinetic/spastic	IV–V	13.75
Choi	Korea	2011	10	Non-specific	Non-specific	2–9
Christiansen	Denmark	2008	24	Non-specific	I–IV	3.167
Grecco	Brazil	2013	33	Non-specific	I–III	3–12
Labaf	Iran	2015	28	Diplegic	Non-specific	2–6
Reddihough	Australia	1998	22	Non-specific	Non-specific	1–3
Scholtes	Netherland	2010	48	Spastic	I–III	6–13.8
Shamsoddini	Iran	2009	24	Spastic/Diplegic	Non-specific	2–6
Shamsoddini	Iran	2010	22	Spastic/Diplegic/Hemiplegic	Non-specific	2–6
Tsorlakis	Greece	2004	34	Hemiplegia/Diplegia/	I–III	3–14
				Quadriplegia

**Table:** 

	**Intervention**		**Dosage**	
**Study**	**TEG**	**SCG**	**TEG**	**UCG**
Arai	Bobath	Conventional	2.4	2.4
Bar-haim	Motor learning coaching	NDT	0.6	0.6
Bower	Aims+Intensive	Aims+Conventional	0.92	0.2
	Goals+Intensive	Goals+Conventional	0.93	0.22
Bryant	Bike/Treadmill	Conventional	0.3	0.3
Choi	Task-oriented approach	NDT	0.5	0.5
Christiansen	Intermittent physical therapy	Conventional	0.24	0.3
Grecco	Treadmill	Overground walking	0.2	0.2
Labaf	NDT	Conventional	0.6	0.6
Reddihough	Conductive education	Conventional	0.56	0.58
Scholtes	Functional strength training	Conventional	0.6	0.6
Shamsoddini	Sensory integration therapy	Conventional	1	1
Shamsoddini	Functional strength training	Conventional	0.6	0.6
Tsorlakis	Intensive NDT	NDT	0.83	0.33

+ *GMFCS; NDT, Neurodevelopmental therapy; TEG, therapeutic exercise group; SCG, standard care group*.

### Overall Improvement

**Figure 2:**
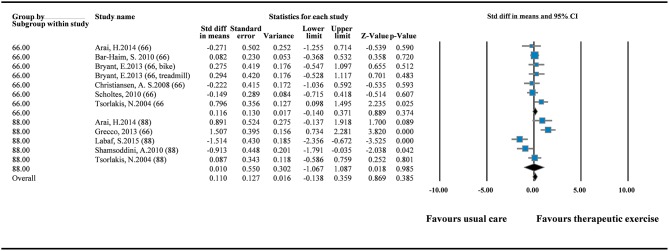
Forest plot of pairwise comparison between therapeutic exercise and standard care (overall GMFM). *I*^2^ = 0%; Q = 10.139; df = 11.

Nine RCTs were pooled to explore the effects of therapeutic intervention on GMFM improvement (12, 15, 16, 18–21, 23, 24). The GMFM-66 was reported in six of the nine RCTs, and the GMFM-88 was reported in five of the nine RCTs. Because two versions of the measurement tool were used in the RCTs, we pooled the data in SMDs. The results of overall pooling demonstrated that in GMFM improvement did not differ significantly between the experimental group that received therapeutic intervention and the control group that received standard care (SMD = 0.110; 95% CI = −0.138 to 0.359; *I*^2^ = 0%). Moreover, no significant differences were observed between the two groups when GMFM-66 (SMD = 0.116; 95% CI = −0.140 to 0.371) and GMFM-88 (SMD = 0.010; 95% CI = −1.067 to 1.087) were used separately (Figure 2). Egger's test results did not indicate small study bias in this pooled analysis (*p* = 0.897; Supplementary File 1).

In one-group meta-analysis, notably, the GMFM-66 showed significant improvement (MD = 1.922; 95% CI = 1.408–2.436; *I*^2^ = 51.334%), when the experimental (therapeutic intervention) group and control (standard care) group were pooled together (Supplementary File 2a). However, the GMFM-88 did not show significant benefits of the therapeutic intervention in the one-group model (MD = 0.127; 95% CI = −4.942 to 5.196; *I*^2^ = 92.075%; Supplementary File 2a). Although no small study bias was detected in this meta-analysis (*p* = 0.998), extremely high heterogeneity was observed among the RCTs using GMFM-88 (Supplementary File 2b).

### Additional Analysis

**Figure 3:**
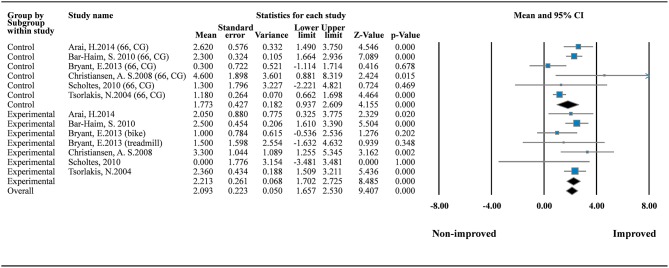
Forest plot of GMFM improvement (the GMFM-66). Control (standard care) *I*^2^ = 68.659%; Q = 15.953; df = 5. Experimental (therapeutic exercise) *I*^2^ = 0%; Q = 5.779; df = 6.

To explore the effects of daily dose and program duration on GMFM scores, we conducted additional analysis of GMFM-66 by using subgroup analysis and metaregression. Subgroup analysis in the one-group model showed that both therapeutic intervention (MD = 2.213; 95% CI = 1.702–2.530; *I*^2^ = 0%) and standard care (MD = 1.773; 95% CI = 0.937–2.609; *I*^2^ = 68.659%) can improve motor function significantly (Figure 3). No small study bias was detected in this meta-analysis of the one-group model (*p* = 0.717; Supplementary File 3a).

**Figure 4:**
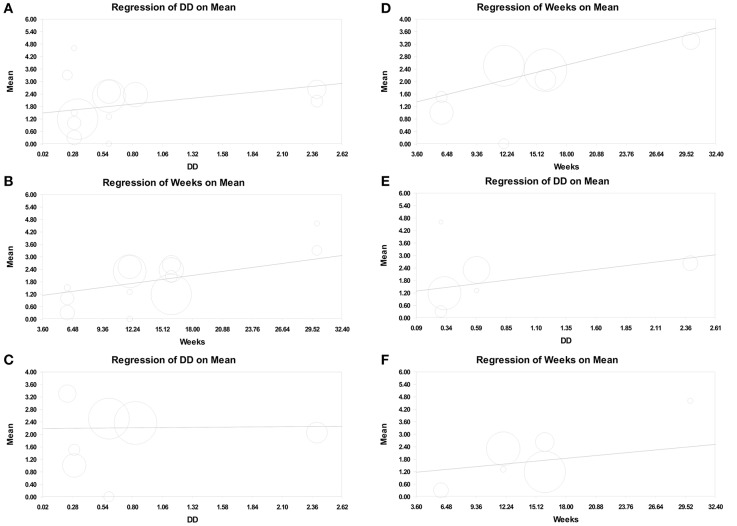
Meta-regression of intensity on GMFM improvement (assessed using GMFM-66, Mean). **(A)** Number of daily training hours (daily dose, DD) on overall GMFM improvement. **(B)** Program duration (weeks) on overall GMFM improvement. **(C)** Number of daily training hours on the GMFM improvement in the therapeutic exercise group. **(D)** Program duration on the GMFM improvement in the therapeutic exercise group. **(E)** Number of daily training hours on the GMFM improvement in the standard care group. **(F)** Program duration on the GMFM improvement in the standard care group.

In metaregression analysis, the roles of daily training hours (h) and program duration (weeks) in improving motor function were explored. The result showed that the number of daily training hours was significantly correlated with motor function improvement when GMFM-66 was used (point estimate = 0.549; 95% CI = 0.050–1.047; *p* = 0.031; Supplementary File 3b). The program duration was also positively correlated with motor function improvement (point estimate = 0.067; 95% CI = −0.007 to 0.140; *p* = 0.075), but the significance of the positive correlation was marginal (Figures 4A,B, Supplementary File 3c).

Moreover, we separated therapeutic intervention and standard care in metaregression analysis. In the experimental (therapeutic intervention) subset, the number of daily training hours was not associated with motor function improvement (point estimate = 0.027; 95% CI = −0.918 to 0.972; *p* = 0.955; Figure 4C, Supplementary File 4a), but the program duration was positively correlated with the improvement; however, the significance of the correlation was low (point estimate = 0.082; 95% CI = −0.015 to 0.179; *p* = 0.096; Figure 4D, Supplementary File 4b). By contrast, in the control (standard care) subset, the number of daily training hours was positively associated with motor function improvement (point estimate = 0.692; 95% CI = 0.100–1.284; *p* = 0.022; Figure 4E, Supplementary File 5a), and the program duration was not correlated with motor function improvement (point estimate = 0.046; 95% CI = −0.066 to 0.159; *p* = 0.419; Figure 4F, Supplementary File 5b).

## Discussion

To our knowledge, our study is the first synthesized evidence relating intensity of common therapeutic exercises and gross motor function improvement. We also provided daily dose (daily training hours) as a novel definition of intensity of physical exercise.

Many factors play crucial roles in enhancing motor functions in children with CP (25, 26). The effectiveness of different types of intervention in patients with CP has been reported in previous systematic reviews. However, most of them did not provide robust results of efficacy because of heterogeneity in participants, type of interventions, intensity, duration of physical rehabilitation program, and small number of participants (8, 9, 27, 28). Intensive physical therapy is considered to be associated with improved outcomes. However, no consensus regarding the optimal intensity of physical training was reached in previous systematic reviews. A possible reason for a lack of consensus was the divergent definition of intensity in previous studies. Divergent definition is a barrier in evidence synthesis and results in non-robust evidence. Furthermore, in physical rehabilitation is inevitable while studying different types of interventions; heterogeneity interferes with data synthesis and comparison. We also faced similar challenges in our systematic review and meta-analysis. Therefore, we attempted to overcome the divergent definition of intensity of physical rehabilitation using daily training hours. In pharmacology, the concept of “defined daily dose” was developed to enable comparisons of drug consumed across different countries. In our study, we used a similar concept in exercise intensity for rehabilitation.

The GMFM is a reliable clinical measurement tool designed to evaluate changes in gross motor function in children with CP. An average change of 1.58 in the score in GMFM-66 has been suggested as reference data for clinically meaningful improvement (29). A systematic review reported that GMFM scores exhibited greater improvement in intensive programs than in non-intensive programs and with intermittent schedules than in continuous schedules (11). Our study showed similar trends. The results of our study showed not only a trend of a gain of >1.5 points after treatment but also a strong relationship between an increase in the number of daily of physical rehabilitation (daily training hours) and gross motor function improvement, whether in intensive or standard therapy. We also demonstrated that physical rehabilitation programs with relatively long durations promote motor development in children with CP. However, we noted that total duration of the physical rehabilitation program significantly affected improvement in motor function in structured therapies, whereas daily training hours exerted a more significant effect than the overall program duration in standard therapy. Considerable statistical heterogeneity was not observed in overall pooling and one-group meta-analysis of the GMFM-66 scores.

The present study had at least three strengths. First, because studies investigating gross motor function and functional skill had different durations and frequencies of rehabilitation sessions, our study proposed a novel idea defining a daily dose in terms of daily training hours to overcome this heterogeneity. Knowledge of the daily training hours can not only enable clinicians to develop practical short-term or long-term intervention programs but also provide an estimate of optimal interventions for rehabilitating patients with CP. In addition, the average defined daily dose of the included therapeutic intervention was 0.752 h/d, and the average total duration of the included therapeutic intervention was 14 weeks. The average defined daily dose of the standard therapy was 0.755 h/d and the average duration of the included standard therapy was 15.3 weeks. Our study provides the aforementioned prescription guidance information to assist clinicians and policy makers. Second, we excluded observational studies, because according to the widely accepted hierarchy of evidence, results of observational studies are more prone to methodological biases than those of RCTs. Third, we excluded exercise requiring unique intervention or equipment to minimize heterogeneity and increase the global generalizability of our results.

## Limitations

Although our study had some strengths, the findings must be interpreted with caution because of some limitations. First, although we excluded the studies that used unique interventions or equipment for rehabilitation, training programs of the intervention and control groups differed in design. For example, therapeutic exercise programs were designed differently in the included RCTs. In the intervention group, the therapeutic exercise program consisted of motor learning coaching, task-oriented treatment, static exercise bike sessions, and treadmill sessions and followed an intermittent schedule with intensive therapy. However, the program designed for the comparison or control group consisted of neurodevelopmental treatment and standard therapy and followed a continuous schedule. Therefore, conceptual heterogeneity was observed between the intervention group and the control group. However, we did not detect significant heterogeneity in our meta-analysis. Although we observed some remarkable correlations between therapeutic exercise intensity and GMFM improvement with low statistical heterogeneity in patients with CP, the findings should be cautiously translated to clinical practice because of conceptual heterogeneity. Second, some of our eligibility criteria resulted in the exclusion some relevant trials. For instance, we excluded numerous studies that did not provide a specific context of rehabilitation. Inclusion of these studies could have altered the trends observed in our study results if the excluded studies had provided clear information about the execution of the reported rehabilitation programs. To increase the global applicability of our results, we excluded studies that reported the use of unique equipment or passive methods in the therapeutic intervention. This criterion limited the applicability of our conclusions to only common rehabilitation programs because we excluded the trials using unique intervention or equipment. In studies that reported the use of unique interventions or equipment for optimal high-intensity exercise, the issue of compliance after the treatment program ends is inevitable. Dunst et al. (30) and Novak (31) have reported that when assigning a home program to enhance treatment dose, either the patient or family will exhibit low compliance. In other words, developing rehabilitation programs that involve unique interventions or equipment should be discussed. Third, the RCTs included in this systematic review investigated a wide range of motor ability in patients with CP. We could not conduct additional analysis because of the paucity and complexity of details of the severity of CP. In clinical practice, patient characteristics, including CP severity, should be considered while designing rehabilitation programs. Finally, the RCTs in our systematic review reported short-term outcomes of GMFM improvement. They reported follow-up durations between 2 and 30 weeks. Few of them showed result of retain test. Long-term effects of therapeutic exercise intensity should be examined by further randomized controlled trials with large sample size.

## Conclusions

In summary, although physical exercise improved gross motor function in patients with CP, the extent of improvement did not differ significantly between therapeutic interventions and standard therapies. Notably, exercise intensity played a crucial role in improving gross motor function in the patients receiving therapeutic interventions and standard therapies. The number of daily training hours of therapeutic interventions did not improve gross motor function; however, the duration of standard therapy programs may have affected improvement in gross motor function. By contrast, the program duration of standard therapies did not improve gross motor function but daily dose (number of daily training hours) improved gross motor function. Therefore, we recommended that program duration and daily dose (number of daily training hours) should be considered while designing rehabilitation programs. However, our study could not provide a threshold for either program duration or daily dose (number of daily training hours). Additional RCTs are warranted for determining the thresholds of program duration or daily dose.

## Data Availability

No datasets were generated or analyzed for this study.

## Author Contributions

C-WH reviewed the literature, collected data, prepared summary tables, and wrote the first draft of the manuscript. Y-NK designed the study, reviewed the literature, performed meta-analysis, interpreted the results, and critically reviewed the manuscript. S-HT reviewed the literature, collected data, interpreted the results, and wrote the first draft of the manuscript.

### Conflict of Interest Statement

The authors declare that the research was conducted in the absence of any commercial or financial relationships that could be construed as a potential conflict of interest.
