# Supplementary material for: Effects of Therapeutic Exercise Intensity on Cerebral Palsy Outcomes: A Systematic Review With Meta-Regression of Randomized Clinical Trials
Source: Front Neurol. 2019 Jun 21;10:657. doi: 10.3389/fneur.2019.00657 (PMC6598595; doi:10.3389/fneur.2019.00657)
Supplement: Supplementary file 1 [file Data_Sheet_1.docx]

Supplementary Material

# Supplementary Figures and Files

## Supplementary Figures

**
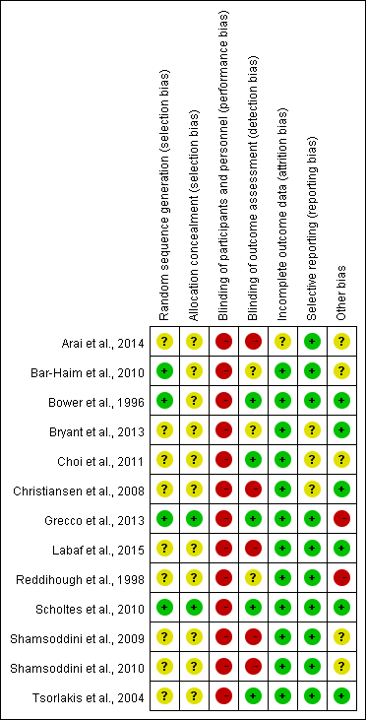
**

**Supplementary Figure 1.** Risk of bias.

## Supplementary Files

**Supplementary File 1.** Small study bias in the overall pairwise comparison of GMFM.


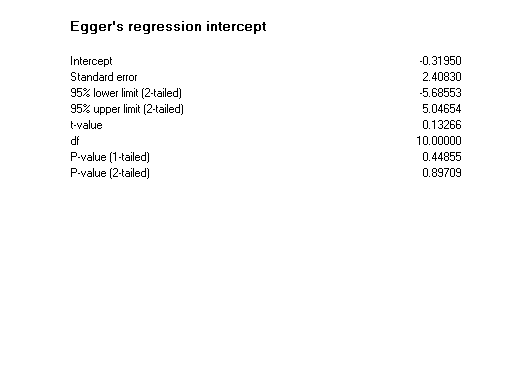


**Supplementary File 2.** GMFM Improvement (Overall).


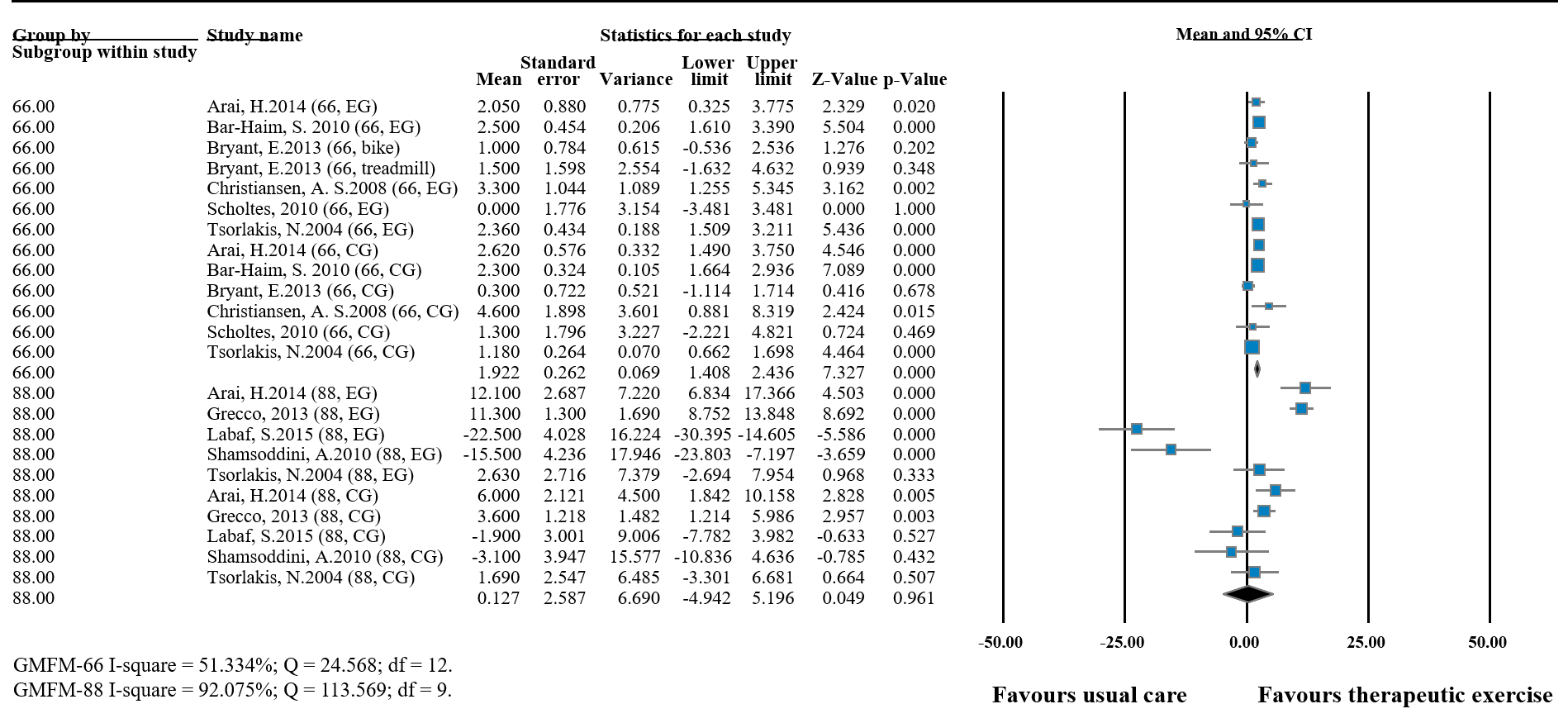
(a)


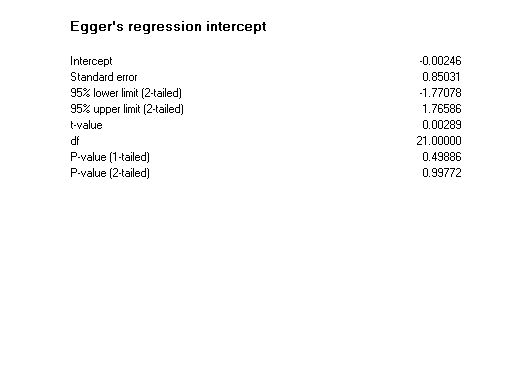
(b)

**Supplementary File 3.** Meta-regression for the GMFM-66 Improvement (Overall).


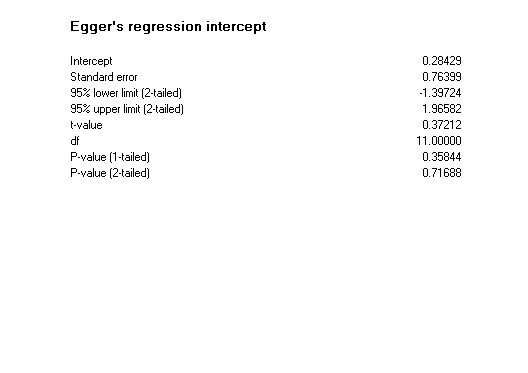
(a)


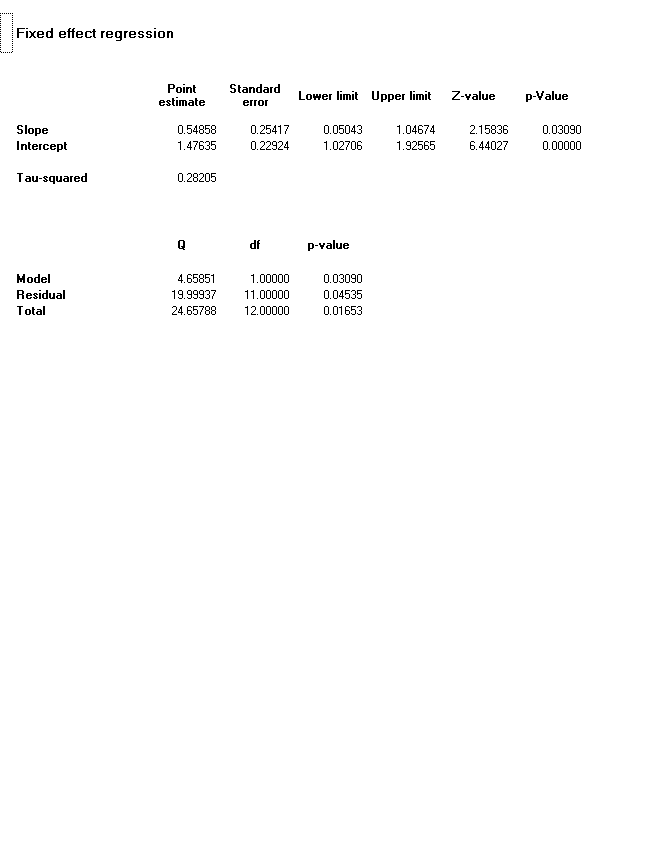
(b)


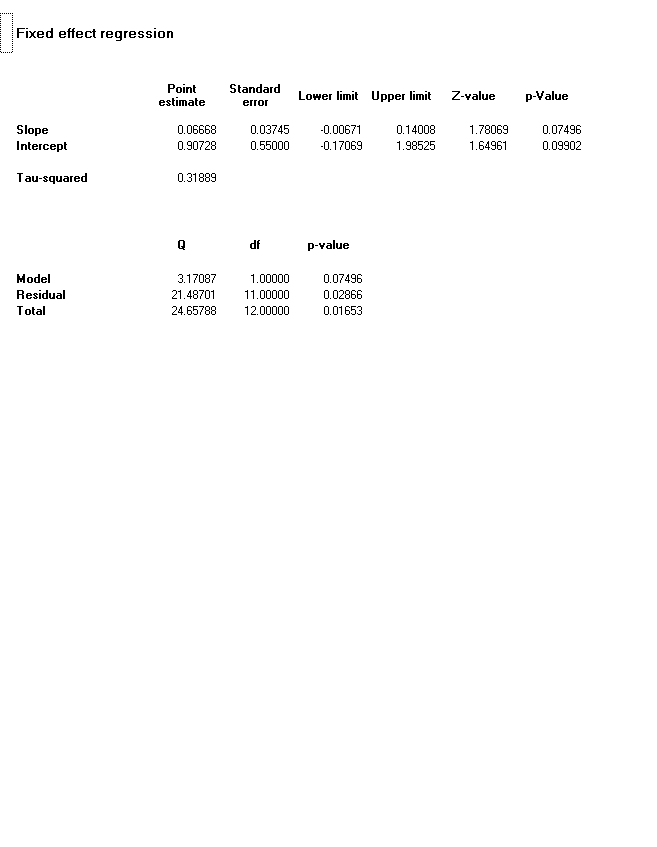
(c)

**Supplementary File 4.** Meta-regression for the GMFM-66 Improvement (Therapeutic exercise).


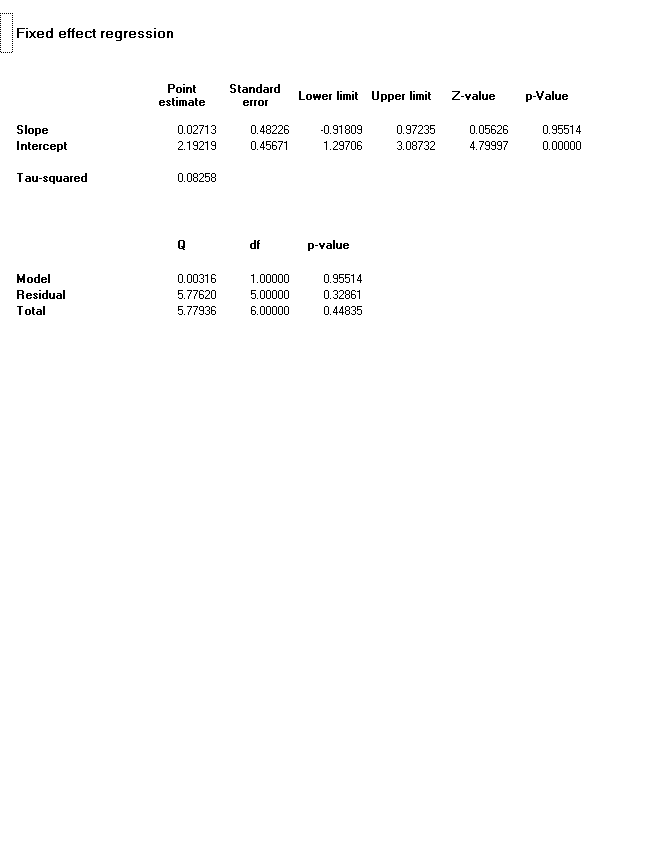
(a)


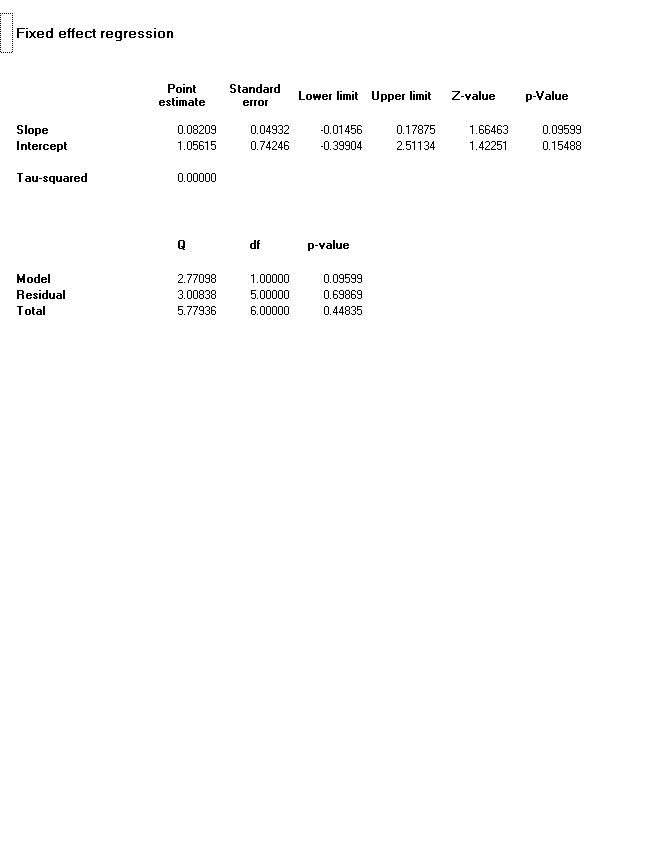
(b)

**Supplementary File 5.** Meta-regression for the GMFM-66 Improvement (Usual care).


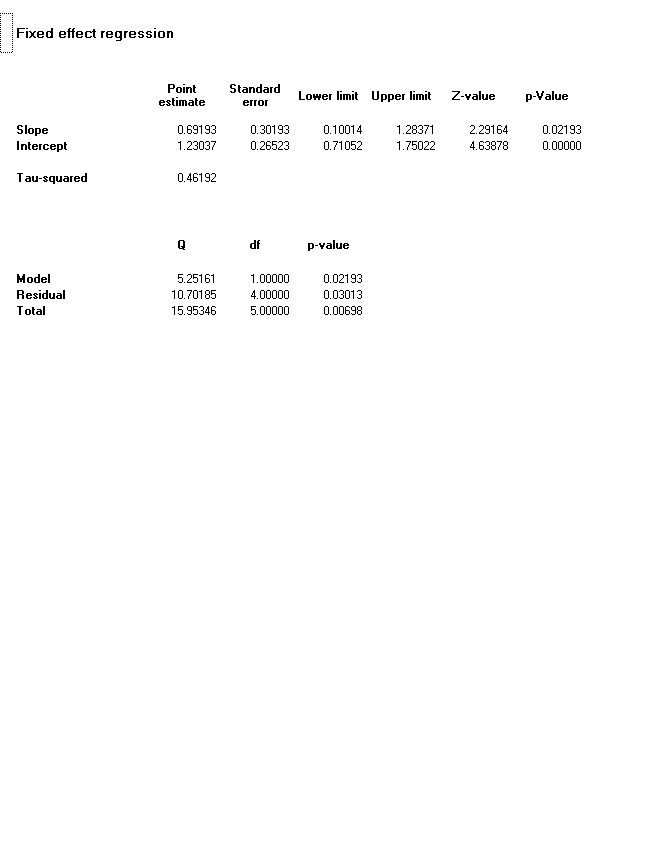
(a)


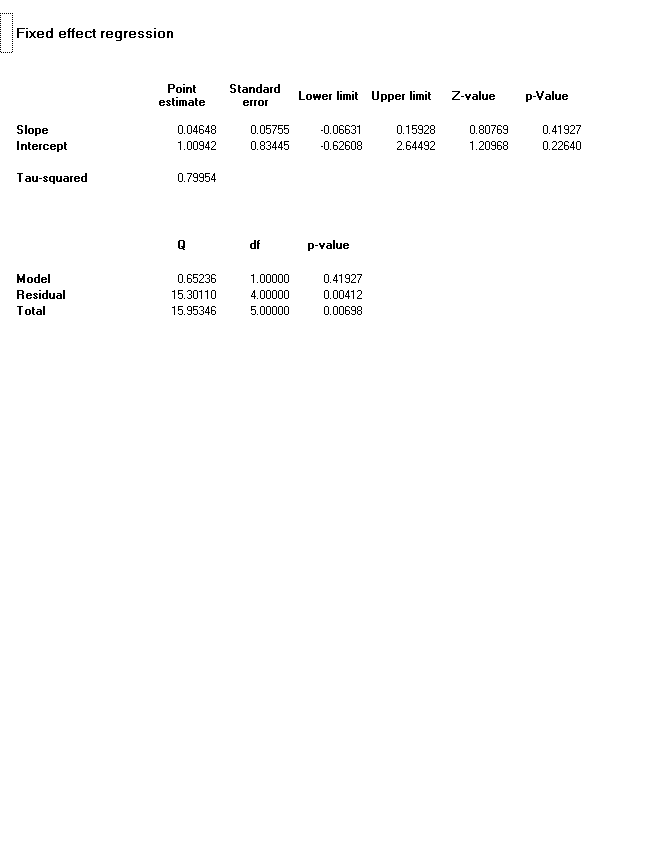
 (b)
